# Supplementary material for: Exposure to Obesogenic Environments during Perinatal Development Modulates Offspring Energy Balance Pathways in Adipose Tissue and Liver of Rodent Models
Source: Nutrients. 2023 Mar 4;15(5):1281. doi: 10.3390/nu15051281 (PMC10005203; doi:10.3390/nu15051281)
Supplement: Supplementary file 1 [file nutrients-15-01281-s001.zip › nutrients-2235413-supplementary.pdf]

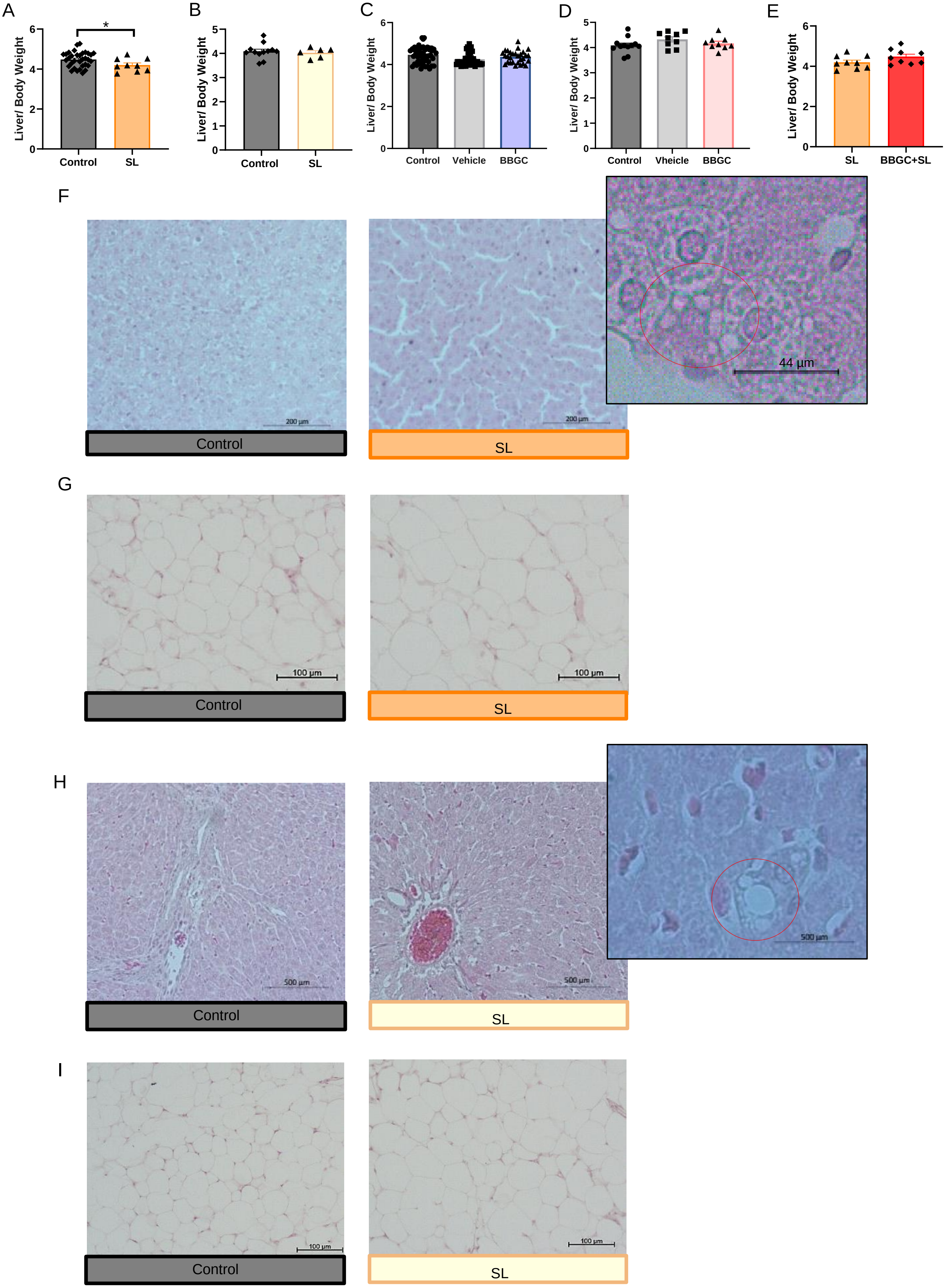


Figure S1 - The liver mass per body weight at PND 45: Male control and SL (A), female control and SL (B), male control, vehicle and BBGC (C), female control, vehicle and BBGC (D) and male SL and BBGC + SL (E). Representative images of the liver and VAT stained with Haematoxylin-eosin (100 x) – liver and VAT from male SL (F and G, repectevely) and liver and VAT from female SL (H and I, respectively).

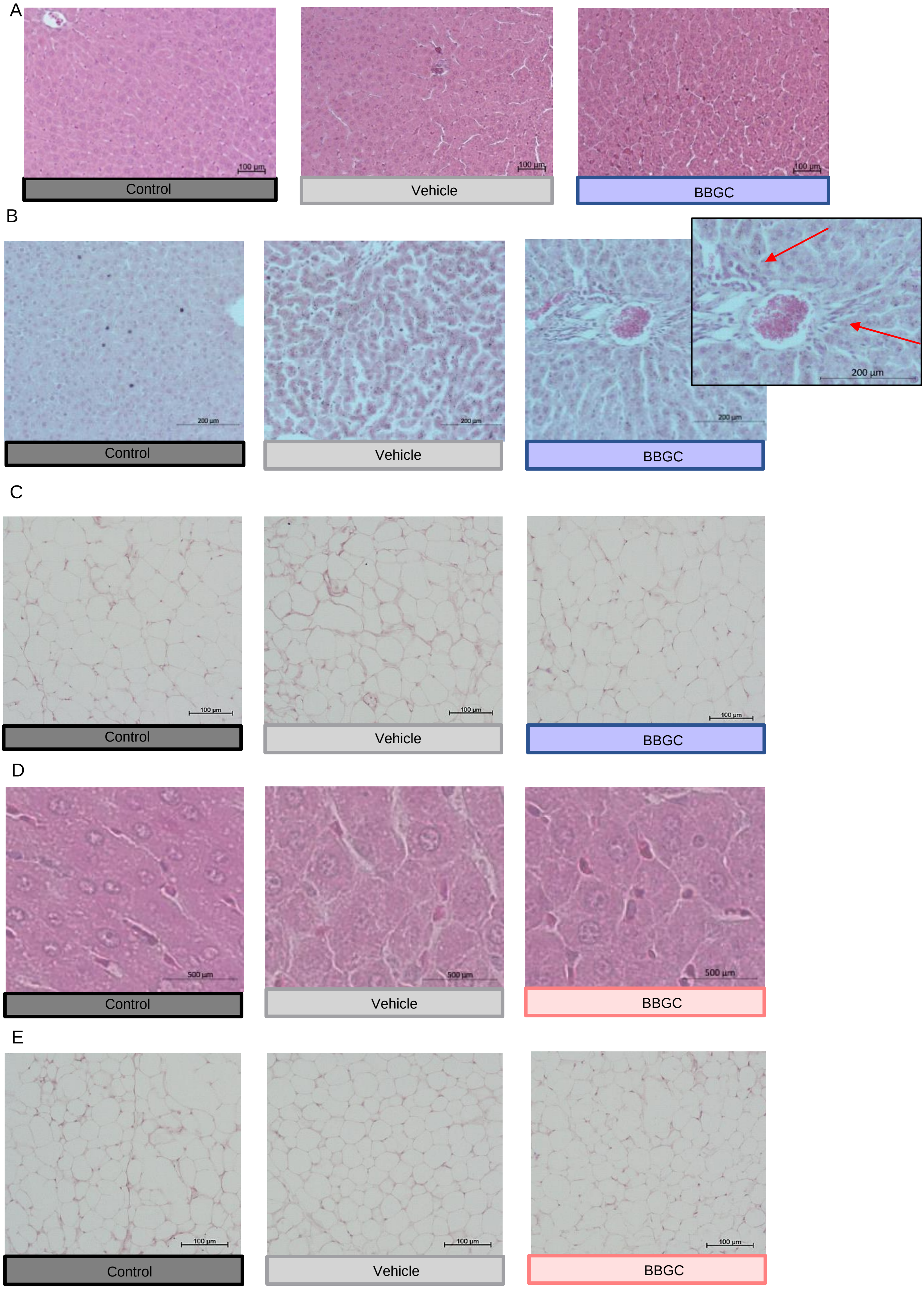


Figure S2 - Representative images of the liver and VAT stained with Haematoxylin-eosin (100 x) – liver from dams treated with BBGC (A); liver and VAT from male BBGC (B and C, respectively) and liver and VAT from female BBGC (D and E, respectively).

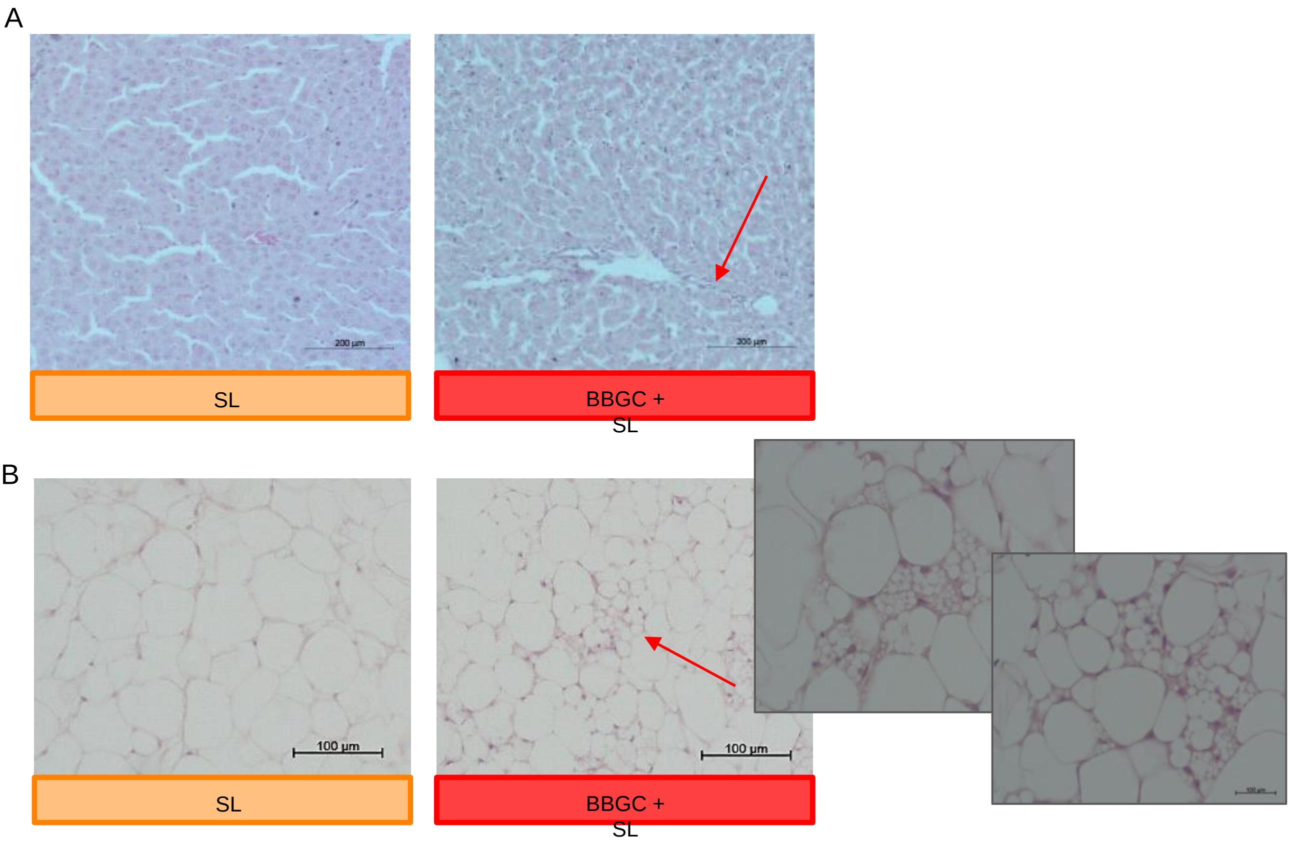


Figure S3 - Representative images of the liver and VAT stained with Haematoxylin-eosin (100 x) – liver and VAT from BBGC-offspring male SL (A and b, respectively).

## Table S1 - Primary antibodies used in Western Blotting

| Antibody | Molecular weight (kDa) | Dilution | Manufacturer | Secondary Antibody | Reference |
|---|---|---|---|---|---|
| Anti-AMPKα | ≈ 62 kDa | 1:1000 | Cell signaling | Anti-Rabbit | 2532 |
| Anti-p(Thr172)-AMPKα | ≈ 62 kDa | 1:1000 | Cell signaling | Anti-Rabbit | 2535 |
| Anti-Calnexin | 83 kDa | 1:2000 | Sicgen | Anti-Goat | AB0037-500 |
| Anti-D1R | ≈ 48 kDa | 1:1000 | Abcam | Anti-Rabbit | ab81296 |
| Anti-GHSR1α | ≈ 42 kDa | 1:500 | Abcam | Anti-Rabbit | ab85104 |
| Anti-Insulin Rβ | ≈ 95 kDa | 1:1000 | Cell signaling | Anti-Rabbit | 3025 |
| Anti-MG-H1 | | 1:1000 | HycultBiotech | Anti-Mouse | HM5017 |
| Anti-NPY1R | ≈ 52 kDa | 1:1000 | Bio-Rad | Anti-Sheep | 6732-0150 |
| Anti-NPY2R | ≈ 52 kDa | 1:1000 | Sicgen | Anti-Goat | AB0328-100 |
| Anti-PPARα | ≈ 54 kDa | 1:1000 | Abcam | Anti-Rabbit | ab227074 |
| Anti-PPARγ | ≈ 54 kDa | 1:1000 | Cell Signalling | Anti-Rabbit | 81b8 |
